# Supplementary material for: Design of Physicochemical Properties of Eggs as a Result of Modification of the Fat Fraction of Laying Feed
Source: Molecules. 2024 Mar 11;29(6):1242. doi: 10.3390/molecules29061242 (PMC10975122; doi:10.3390/molecules29061242)
Supplement: Supplementary file 1 [file molecules-29-01242-s001.zip › molecules-2892481-supplementary.pdf]

Design of physicochemical properties of eggs as a result of modification of the fat fraction of laying feed.

- Supplementary materials

Agnieszka Filipiak-Florkiewicz, Maja Dymińska-Czyż , Beata Szymczyk, Magdalena Franczyk-Żarów, Renata Kostogrys, Adam Florkiewicz and Marcin Lukasiewicz

Table S1. Composition of lying hens feed

Item	g/kg
Wheat	255
Maize	345
Soybean meal (460 g/kg crude protein)	217
Dried grass	30
Limestone	85
Dicalcium phosphate	17
Sodium chloride	3
Vitamin and mineral premix[1]	5
DL-Methionine	1
L-Lysine hydrochloride	2
Rape	40
**Calculated nutrient content (g/kg)**	
Metabolizable energy (MJ/kg)[2]	11.55
Crude protein	169.2
Crude fibre	3.7
Methionine + Cystine	7.4
Lysine	8.4
Sodium	1.7
Calcium	36.5
Chloride	1.7
Available phosphorous	6.8

[1]The premix provided per 1 kg of diet: vitamin A—10,000 IU (retinol); vitamin D3—2000 IU (cholecalciferol); vitamin E—20 IU (dl-alpha-tocopherol); vitamin K3—1.5 mg (menadione); vitamin B1—1 mg (thiamine); vitamin B2—4 mg (riboflavin); vitamin B6—1.5 mg (pyridoxine); vitamin B12—0.02 mg (cyanocobalamin); biotin—0.05 mg Ca-pantothenate—8.7 mg; niacin—20 mg; folic acid—0.8 mg; choline chloride—200 mg; manganese—85 mg; zinc—60 mg; iron—45 mg; copper—8 mg; iodine—1 mg; selenium—0.25 mg; [2]Calculated acording to Janssen, W.M.M.A. European Table of Energy Values for Poultry Feedstuffs, 3rd ed.; Subcommittee Energy of the Working Group nr. 2 Nutrition of the European Federation of Branches of the World's Poultry Science Association: Beekbergen, The Netherlands, 1989; ISBN 90-71463-00-0. [Google Scholar] [3] Experiment I A- control group (rapeseed oil 2.5%, linseed oil 1.5%);, B - rapeseed oil 2.0%, linseed oil 1.5% and pomegranate seed oil 0.5%; C - rapeseed oil 1.5%, linseed oil 1.5% and pomegranate seed oil 1.0%;D - rapeseed oil 1.0%, linseed oil 1.5% and pomegranate oil 1.5%.

Experiment II E - control group (rapeseed oil 4%); F - rapeseed oil 3.5% and pomegranate oil 0.5%; G - rapeseed oil 3.0% and pomegranate oil 1.0%; H - rapeseed oil 2.5% and pomegranate oil 1.5%.

Table S2. Fatty acid profile of oils used in all experiments, %

Fatty acids	RO	LO	PSO
C 12:0	-	-	0.03
C 14:0	0.20	0.05	0.06
C 16:0	4.40	6.5	6.35
C 17:0	0.04	0.05	0.09
C 18:0	2.0	3.5	3.36
C 18:1	60.5	18.1	16.23
C 18:2 n-6	20.1	15.0	13.74
C 18:3 n-3	11.8	56.78	0.41
C 20:0	0.66	0.02	0.70
C 20:1	-	-	0.96
C 24:0	0.30	-	0.20
CLnA	-	-	58.83
SFA	7.60	10.12	10.77
MUFA	60.50	18.10	15.59
PUFA	31.90	71.78	73.0

Table S3. The significance of the differences; p-values – Experiment 1

**Parameter**	**p-value**
Egg white pH	0.0316
Egg white index	0.0360
ΔE[1]	0.0436
Dry matter:	
Y[2]	0.0246
W[3]	0.0001
Protein:	
W	0.0000
Ash	
C14:0 tetradecanoic (myristic acid)	0.0283
C16:0 hexadecanoic (palmitic acid)	0.0460
C18:0 octadecanoic (stearic acid)	0.0009
C16:1 *trans*-3-hexadecenoic	0.0200
C18:1 *cis*-9-Octadecenoic (oleic acid)	0.0016
C18:2 *n*−6 *cis,cis*-9,12-octadecadienoic (linoleic acid)	0.0139
C18:2 Conjugated linoleic acids - CLA	0.0000
C18:3 *n*-3 *cis,cis,cis*-9,12,15-octadecatrienoic (α-linolenic acid)	0.0002
C18:3 Conjugated linolenic acid - CLnA	0.0008
C20:4 *n*-6 5,8,11,14-*all-cis*-eicosatetraenoic (arachidonic acid)	0.0027
C22:6 *n*-3 docosahexaenoic - DHA (cervonic acid)	0.0466

[1]-color difference
[2]Y – egg yolk,
[3]W – egg white

Table S4. The significance of the differences; p-values – Experiment 2

**Parameter**	**p- value**
Egg yolk index	0.0000
Egg white index	0.0005
ΔE[1]	0.0065
L[2]	0.0113

a[3]	0.0000
Dry matter:	
Y[4]	0.0498
W[5]	0.0011
Protein:	
Y	0.0231
W	0.0000
Ash:	
Y	0.0351
C14:0 Tetradecanoic (myristic acid)	0.0080
C18:0 Octadecanoic (stearic acid)	0.0128
C14:1 9-tetradecenoic (myristoleic acid)	0.0056
C16:1 *trans*-3-hexadecenoic	0.0006
C18:1 *cis*-9-Octadecenoic (oleic acid)	0.0006
C16:2 Hexadecadienoic acid	0.0018
C18:2 *n*−6 *cis,cis*-9,12-octadecadienoic (linoleic acid)	0.0043
C18:2 Conjugated linoleic acids - CLA	0.0000
C18:3 *n*-3 *cis,cis,cis*-9,12,15-octadecatrienoic (α-linolenic acid)	0.0047
C18:3 Conjugated linolenic acid -CLnA	0.0005
C20:2 eicosadienoic	0.0100
C20:3 *n*-6 *cis,cis,cis*-8,11,14-eicosatrienoic dihomo-γ-linolenic acid	0.0056

[1]-color difference
[2]-luminance
[3]-red/green value
[4]Y – egg yolk,
[5]W – egg white
